# Chiropractic Treatment of Dairy Cows

**DOI:** 10.3390/vetsci11110570

**Published:** 2024-11-15

**Authors:** Franziska C. Wagner, Felicitas Hesse, Johanna Wehrle, Christoph K. W. Mülling

**Affiliations:** Institute of Veterinary Anatomy, Histology and Embryology, Faculty of Veterinary Medicine, Leipzig University, 04103 Leipzig, Germany

**Keywords:** anatomy, dairy cow, lameness, manipulation, musculoskeletal, spine

## Abstract

Lameness, also attributed to musculoskeletal disorders of the back, is a major issue in dairy cow farming, affecting both animal welfare and economics. Chiropractic techniques are well studied in humans and horses for the treatment of musculoskeletal disorders. The anatomy of the bovine spine differs significantly from that of horses, but the knowledge of the animal’s specific anatomy is crucial for chiropractic treatment. This report explores how chiropractic methods could be adapted to cattle, considering their unique anatomy, and potentially integrated into standard veterinary care. Five case reports and an example of measuring the alignment of the facet joints in the spine are presented and discussed. Chiropractic treatments showed success in alleviating lameness and spinal issues in dairy cows, particularly when combined with other interventions. However, the role of chiropractic treatment in cattle as a complementary therapy needs further research.

## Simple Summary

Lameness, which can also result from back problems, is a major issue in dairy cows. It affects both the well-being of the cows and the farm’s economy. Chiropractic techniques, as forms of manual therapy, are well known for treating back problems in humans and horses. The cow’s spine is quite different from that of the horse, but understanding cow anatomy is crucial for effective chiropractic treatment. This report examines how chiropractic methods can be adapted to help cows based on their unique anatomy. It also presents five cases and gives an example of measuring the angulation of spinal joints. Chiropractic treatments helped reduce lameness and back problems in dairy cows, especially when used in conjunction with other treatments. However, more research is needed to fully understand the role of chiropractic care in cows.

## 1. Introduction

Lameness is one of the most important problems in dairy cow farming [1]. The resulting animal welfare and economic issues are well documented [2], but herd prevalence remains high [3]. Musculoskeletal injuries, including disorders of the leg, hip, and back, were categorised as belonging to class III of IV in terms of disability weight in a study by McConnel et al. [4].

Chiropractic treatment aims to improve the function of the musculoskeletal and nervous systems and their effect on general health [5,6]. The effectiveness of chiropractic manipulation is well studied in the treatment of human [7,8,9,10,11] and equine [12,13,14,15] back pain. Despite its potential benefits, there is limited scientific evidence supporting its efficacy in veterinary medicine, and there are almost no data for cattle [16]. To the authors’ knowledge, only one case report of chiropractic practices in cattle has been published [17].

Chiropractic practice involves spinal manipulative therapy, which requires a thorough understanding of spinal anatomy, biomechanics, and pathology [18]. Chiropractic mobilisation and manipulation are manual therapy techniques used to treat musculoskeletal disorders [19]. Mobilisation techniques involve the application of low-velocity, controlled movements to joints and surrounding tissues to improve mobility and reduce pain [20]. Chiropractic manipulation, also known as spinal manipulation or chiropractic adjustment, means the application of a high-velocity, low-amplitude thrust to a specific joint, typically the spinal vertebrae, to restore its normal function and alleviate pain [21]. The technique is based on the principle that misalignments or dysfunctions in the spine can affect the nervous system and general health. By correcting these misalignments, chiropractic manipulation aims to improve joint mobility, reduce pain, and enhance the body’s natural healing processes [22]. For any chiropractic manipulation, the line of correction is crucial: it ensures that the force applied during the adjustment is precisely directed to achieve the desired therapeutic outcome and restore joint function [21]. The line of correction, which refers to the angle of the force applied during a manipulation, is determined based on the joint’s anatomy and biomechanics. Accurate application reduces the risk of injury or adverse effects by preventing surrounding tissues and structures from experiencing undue stress [23].

One of the questions underlying this case report was the extent to which chiropractic treatment needs to be adapted to the specifics of bovine anatomy. In addition, the procedure for chiropractic treatment in cattle is presented with regard to species-specific handling. Finally, the extent to which chiropractic care can be integrated into the usual medical care of cattle is discussed.

## 2. Anatomic Characteristics of the Bovine Spine

The following is a discussion of the anatomical peculiarities of cattle compared to horses. The focus here is on structures and characteristics that are important for manipulations of the spine. The description moves from cranial to caudal areas.

The occipital bone of the skull articulates with the atlas, with obliquely positioned occipital condyles [24]. The atlas has shorter and thicker wings, with a flatter fossa atlantis than in the horse [24]. Its dorsal (and ventral) tubercle can be divided into two parts [25]. The axis has a higher, almost straight spinous process which increases in thickness caudally but remains undivided [24]. Unlike in the horse, the caudal articular processes in cattle are clearly separated from the spinous process [24] and are structured as independent articular surfaces [26].

Bovine cervical vertebrae (CV) are shorter than equine ones but have longer spinous processes and more massive transverse processes [24]. The spinous processes increase in length with the CV and are obliquely directed craniodorsally from the 3rd to the 6th CV. It is vertical at the 7th CV and measures at about 14 cm [24], which is about twice as long as that of the 6th CV [27]. At the 3rd and 4th CV, it is notched [25,27]. The transverse processes are split as in horses, but they have shorter dorsoventral tubercles and a thicker and elongated cranioventrally directed tubercles [24,27]. The latter increases in size until the 5th CV, then becomes an extensive, ventrally descending plate on the 6th CV, and is completely absent on the 7th CV [24,25,27]. The cranial and caudal articular processes are small compared to those of the horse [25].

The 13 thoracic vertebrae (TV) are longer than in the horse; the shortest is the 6th or 7th TV [24,27]. The first palpable spinous process is that of the 1st TV, which lies cranial to the scapula [28]. The spinous processes of the TV are broader, and therefore nearer together [24], and they are higher than in the horse. They increase in length up to the 3rd and in width up to the 5th (6th) TV, and then decrease progressively [24,27]. From the 12th (13th) TV onwards, the spinous processes are of the same height [26]. This, and the absence of withers [24], are clearly seen in the very straight topline [28]. The spinous processes of the first 5 TV are steeper in their dorsal half [24], and from the 7th to the 11th TV, they are inclined caudally [25], with the spinous process of the 10th TV being the most oblique [24]. The (12th) 13th TV is the anticlinal vertebra [24]. The cartilage caps of the spinous processes of the 1st to the 5th TV ossify by the age of eight years [25,27]. The joint surfaces of the first 11 TV are tangential, and on the last two they are sagittal [27]. Here, the articular processes increasingly form a cylindrical shape [24]. In the last two TV, the cranial mammillary processes are fused with the cranial articular processes and are always fused in the lumbar vertebrae (LV) [25,27]. The intervertebral foramen of the TV, and often also of the first three LV, is usually divided by a bony bar that encloses the caudal vertebral notch [27].

Also, the bodies of the 6 (occasionally 7 [27]) lumbar vertebrae are longer and have relatively flat vertebral arches compared to those of the horse [24]. The intervertebral foramina are wide, especially in the sacral half [24]. The spinous processes are as high as they are wide and decrease caudally in height [24]. The sharp-edged costal processes increase in length and width until the 4th or 5th LV and then decrease; at the 6th LV, they are sometimes split into two endings [24]. The complete congruence of the cylindrical (caudal) or semi-tubular (cranial) articular processes and narrow joint capsules severely restricts movement [27,28]. In general, the bovine spine is much stiffer, which can also be explained by the shortness of the intervertebral discs, which only make up 10% of the total length of the spine [28]. There are no lumbar or lumbosacral intertransverse joints, which are found in the horse [27].

The 5 sacral vertebrae (SV) are fused in the third or fourth year of life (compared to the fourth or in the fifth year of life in a horse) [27]. The sacrum is more or less dorsally curved [24,27] depending on the breed [25]. Its wings are rectangular [24,27], with caudodorsally faced articular surfaces [25,27]. The sacral spinous processes are less high than in the horse and are fused to the middle sacral crest [24]. This is occasionally interrupted between the 4th and the 5th SV [25].

There are 18 (16) to 20 (21) long and strong coccygeal vertebrae (CCV). Among these, the first 5 show more of the usual characteristics of a vertebra than the horse, e.g., a narrow vertebral arch [24] with a vertebral canal [25]. From the 1st (2nd) to the 13th CCV, two haemal processes are formed at the cranial end. These build up a closed haemal arch from the 2nd (3rd) to the 4th (5th) CCV, and otherwise form a caudally decreasing groove [24,27]. The transverse processes are very wide, short, and rectangular at the 1st CCV and decrease in size at the following CCV until their disappearance at the 9th CCV [24]. The cranial articular processes, which mostly do not exhibit an articular surface, are palpable as small humps until the 8th CCV and as weak bumps until the 13th CCV [24].

The thorax consists of eight sternal and five asternal ribs [24]. In cattle, the caudal articular facet of the head of the ribs does not fuse with that of the tubercle (which is the case for the last three ribs of the horse), resulting in less freedom of movement of the caudal ribs [27]. In horses, the joints between the ribs and their cartilage are generally symphyseal; in cattle, the 2nd to 10th ribs have a tight joint [29], but this can also be a synovial joint [27,29]. The cartilage of the last sternal (8th) rib is sometimes articulated with that of the 7th rib [27].

The sternum is slightly curved upwards dorsally, depending on the breed; there is no sternal crest and the xiphoid is smaller than in the horse [24]. The body of the sternum is plate-shaped, whereas in the horse it is keel-shaped [27]. The outer surface of the sternal vertebrae is connected by a membrane [27].

The manubrium is connected to the body by a synovial joint (in horses, it is connected by subsequent ossifying synchondrosis), which functions as an alternating joint [26,27] and allows slight lateral movements [27]. A ligament passes through this joint, connecting the right and left cartilage of the second ribs [29]. The second pair of ribs shares a joint capsule with the manubriosternal joint [27]. The first pair of ribs articulates with two separate joints, whereas in the horse it articulates with one joint at the manubrium [27].

## 3. Exemplary Description of the Bovine Facet Joints

**Figure 1:**
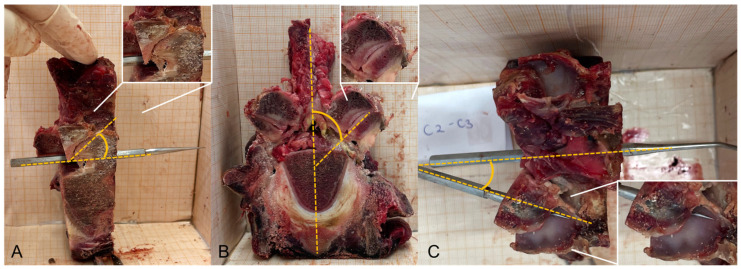
Photographs and measurements of the facet joint inclination (measured angles are indicated with the yellow lines) from (**A**) lateral (*x*-axis), (**B**) cranial (*y*-axis), and (**C**) dorsal (*z*-axis) perspectives of the facet joint between C2 and C3. A detailed view of the facet joint is provided within the images.

To improve our understanding of the inclination of bovine facet joints and thereby enhance our ability to apply the correct line of correction, the vertebral column of an adult cow was obtained from a slaughterhouse. To measure the inclination of the facet joints, the musculature around the spine was removed. The vertebral bodies were sawn through vertically, so that the articular surfaces could be photographed from craniocaudal, laterolateral, and dorsoventral perspectives in a box lined with millimetre paper. The angles on the left side were measured using ImageJ 1.53t (Rasband, W. S., U. S. National Institutes of Health, Bethesda, MD, USA) (Figure 1). Angle (A) was taken from a lateral (*x*-axis) perspective and angle (C) was taken from a dorsal view (*z*-axis). This refers to the spinal canal. Angle (B) describes the facet joint’s inclination around a vertical axis through the spine (*y*-axis). The coordinate system is described according to the work of Cotterill et al. [30], but the definition of positive and negative rotation around the axis is defined from a practical point of view of performing chiropractic therapy on bovines.

The presented measurement is merely exemplary and cannot be generalised. It shows that there are different inclinations of the facet joints along the bovine spine. Studies with a larger number of animals and standardised measurement methods are necessary to be able to make reliable statements. In order to describe the tendencies of the inclination, the angles are rounded to the nearest five. According to the measurements presented in Table 1, the average values of angles from C2/C3 to C6/C7 on the *x*-, *y*-, and *z*-axes are 20°, 50°, and 30°, respectively. In the cervicothoracic transition zone, the average values of angles on the *x*-, *y*-, and *z*-axes are 20°, 65°, and 15°, respectively. In the thoracic spine, the average values of angles on the *x*-, *y*-, and *z*-axes are 10°, 50°, and 15°, respectively. In the lumbar spine, the average values of angles on the *x*-, *y*-, and *z*-axes are 20°, 30°, and 25°, respectively.

## 4. Case Reports

**Figure 2:**
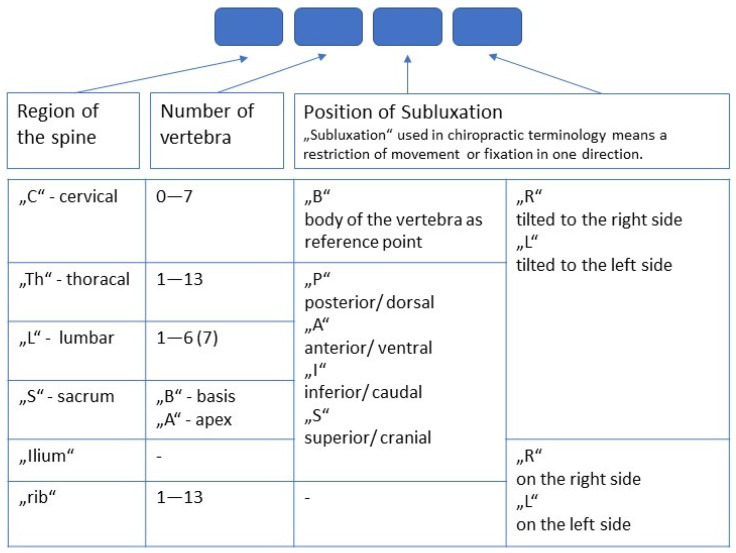
Chiropractic listing system.

In the following, five cases of dairy cows with musculoskeletal disorders that received chiropractic treatment are described. The locomotion is described with a numerical rating system with 0.5-point increments (locomotion score, LS) according to the work of Flower and Weary [31]. The chiropractic findings are described using the Gonstead Listing System, a method commonly used in veterinary chiropractic to describe restrictions in joint motion (Figure 2). The chiropractic findings were addressed with mobilisation and/or manipulation (adjustment) and via myofascial release techniques. For this purpose, the cows were fastened in the feed fence. To treat the spine along the correct line of correction, a treatment box was used.

### 4.1. Cases

#### 4.1.1. Case 1

It was a 6-year- and 4-month-old dairy cow in her 5th pregnancy (58th day of gestation). Following the third treatment, single doses of ketoprofen, vitamin B complex, and vitamin D3 were recommended. These was administered by the veterinarian (Table 2).

#### 4.1.2. Case 2

It was a 5-year- and 9-month-old dairy cow in her 5th pregnancy (158th day of gestation) (Table 3).

#### 4.1.3. Case 3

It was a 3-year- and 11-month-old dairy cow in her 3rd pregnancy (102nd day of gestation). Following the second treatment, single doses of ketoprofen, vitamin B complex, and vitamin D3 were recommended. These were administered by the veterinarian. On the fourth and fifth treatment, tense muscles were massaged, and manual lymph drainage was carried out in addition to the traditional chiropractic treatment (Table 4).

#### 4.1.4. Case 4

It was a two-year- and 7-month-old dairy cow in her 3rd pregnancy (pregnancy examination pending) (Table 5).

#### 4.1.5. Case 5

It was an 8-year- and 7-month-old dairy cow (Table 6).

## 5. Discussion

Veterinary chiropractic care is a growing field, but it still remains under-researched [18,32]. Chiropractic treatments of animals can relieve pain, improve mobility, and promote functional recovery in animals with neuro-musculoskeletal and neurological disorders [18,33]. Their effectiveness is shown in case reports and studies on dogs [34,35] and horses [12,13,14,15], especially for back pain, disc disorders, and neuropathic pain. However, to the authors’ knowledge, there is only one case report on chiropractic treatment in a cow [17]. The limited research on the chiropractic treatment of cattle and other farm animals makes it difficult for veterinarians to recommend or justify chiropractic treatment as a reliable option for farm animals, as there is insufficient evidence of its effectiveness. In livestock, treatments are usually evaluated based on their impact on productivity—such as improvements in milk yield, growth rates, or reproductive success—and their impact on animal welfare. There is a lack of research into whether this form of treatment provides measurable benefits in these key areas. Given the low profit margins in agricultural production, it is unlikely that a treatment will be widely adopted without clear evidence of its efficacy and economic value.

In order to transfer chiropractic techniques to other species, it is important to know their anatomical and biomechanical characteristics. In cattle, the protuberantia intercornualis can be used as a contact zone for the manual treatment of the atlantooccipital joint instead of the flat protuberantia occipitalis. The transverse processes of the lumbar vertebrae, which extend far to the lateral side, can also be used as a contact zone. As the inclination of the facet joints changes along the spine (Table 1, Figure 2), and therefore their biomechanical load also changes, the line of correction must be adjusted accordingly during spinal manipulation.

The measurements of the facet joint angles in three planes, presented here, and the results of Cotterill et al. (1986) on Th6, Th12, and L3 in two planes [30] are insufficient to enable conclusions to be drawn regarding the entire population. The coordinate system was defined according to the work of Cotterill et al., with the *y*-axis in a caudocranial direction, the *x*-axis in a laterolateral direction, and the *z*-axis in a dorsoventral direction. Cotterill et al. examined the superior (cranial) facet joints of ten 6–8-week-old calves. The measurements presented here were performed on an adult specimen. On Th6, Cotterill et al. found an almost vertical alignment (−82.9° +- 5.7 on the *x*-axis) and discovered a lateral tilt of about −27.3° ± 8.4 on the *y*-axis, with a slightly backwards orientation [30]. The deviation from the *y*-axis in the measurements presented here was about 43°. On Th12, Cotterill et al. found an inclination of −70° ± 3.9 on the *x*-axis [30], whereas the measurements presented here displayed an almost vertical alignment of 9°, similar to the published value, on Th6. According to Cotterill et al., the inclination on the *y*-axis is −15.9° ± 7.9 [30]. On L3, the measurements of Cotterill et al. revealed again an almost vertical alignment of −87.2° ± 4.2 on the *x*-axis [30], whereas the measurement presented here showed an inclination of 24°. On the *y*-axis, both working groups found similar tilt values of 27.9 ± 8.0 [30] and 24°. The differences could be due to measurement errors from an insufficient measurement technique, individual characteristics, or breed-related differences. Further research is required to clarify the variations in bovine facet joint inclination. In the meantime, the careful palpation of movement is required to determine the individual inclination of the facet joint being treated.

To perform motion analysis, knowledge about the normal spine mobility is required. In cattle, the largest mobility in the trunk segment is described for areas cranial to Th13 [36]. A further increase in mobility was described in the lumbosacral joint [37]. Wilke et al. specified this through the examination of the thoracic and lumbar spine of a calf [38]. In their study, sagittal bending was shown to increase from Th6 to L6 (around 4° at Th6 to 10.4° at L6), lateral bending had two regional maxima (5.2° at Th9–10 and 6.7° at L2–3) and two minima (4.4° at Th7–8 and 4.2° at Th12–13), and axial rotation in the thoracic spine was about 4.5°, while in the lumbar spine and caudal thoracic vertebrae it was around 1.1°. Th12 is identified as a transitional vertebra [39].

The present case reports show different chiropractic treatment approaches and their varying efficacy in the treatment of spinal problems and lameness in dairy cows. The response of the cows to chiropractic treatment varies greatly depending on the initial findings. In cases 1 to 4, a significant improvement in the symptoms or the absence of symptoms was achieved. In some cases, a combination of chiropractic, mobilisation, and massage was used; in case 3, manual lymph drainage was also performed. In cases 1 and 3, anti-inflammatory medication and vitamin supplements were also used, which appeared to have a supportive effect. However, it remains unclear whether the improvements were due to the chiropractic treatment alone or whether the complementary medication played an important role. The cows showed an involuntary extension of the hind limb when standing and walking. This appeared to be associated with painful processes in the spinal column and disappeared in the course of treatment. Similar behaviour occurs in claw diseases [40] or in bovine spastic syndrome [41]. The former can be excluded due to regular claw control. The cramps in bovine spastic syndrome are more prolonged and last 15–30 s, even in the mild initial phase [42].

The treatment intervals and the number of treatment sessions required varied greatly between the cases, which indicates that the therapy was individually adapted. In most cases, a gradual improvement was observed, which occurred faster in some cows (e.g., case 2) than in others (e.g., case 1) according to the initial findings. Case 5 had a pelvic obliquity that could not be corrected; the symptoms could only be alleviated but not eliminated. In summary, the case reports show that chiropractic treatment can be a valuable therapeutic option for dairy cows with spinal and limb problems, but that it is dependent on the individual problem. In some of the presented cases (1 and 3), access to pasture and combination with other therapeutic measures probably contributed to the improvement of symptoms. A multidisciplinary approach could be helpful.

Often, the detection of lameness does not necessarily lead to treatment, which could be due to the underestimation of the problem or due to financial or management reasons [43]. Lameness leads to unphysiological postures of the back [44], which cause additional pain and movement restriction. Lameness results in significant economic losses in cattle farming due to a combination of direct costs and indirect effects on productivity [45]. A human clinical trial concluded that chiropractic treatment, in addition to usual medical care, significantly improved pain intensity, fitness for work, and the satisfaction of patients with back pain [46]. In this paper, chiropractic treatment was also partially combined with the administration of non-steroidal anti-inflammatory drugs (case 1 and 3). Medication-free lameness management is particularly important in organic farms [47], which highlights the potential use of manual therapy, such as chiropractic treatment. A meta-analysis of risk factors for lameness in dairy cows identified the first 120 days of lactation, large herds, and higher parity [48], which is why we recommend monitoring these cows specifically and possibly presenting them to a veterinarian or manual therapist as a preventive measure.

## 6. Conclusions

The authors intended this case report to stimulate research into evidence-based chiropractic care, and to present further treatment options within the herd management. Both the analysis of a larger number of cases and the conduct of clinical trials are desirable to validate its effectiveness. Chiropractic treatment can be integrated into herd management and can be provided by certified veterinary chiropractors in an integrative (not complementary) manner in relation to conventional medical care. In this study, a gradual improvement was generally observed, although this varied between cases depending on the initial findings. Chiropractic treatment can relieve pain without the waiting period required by many medications. It can promote healing and improve performance parameters and cow comfort. This is desirable from an economic and animal welfare perspective.

## Figures and Tables

**Table 1:** The inclination of the facet joints along an adult bovine spine.

Facet Joint	*x*-Axis	*y*-Axis	*z*-Axis
C1/C2	-	14°	65°
C2/C3	30°	44°	20°
C3/C4	16°	46°	36°
C4/C5	25°	57°	21°
C5/C6	19°	50°	33°
C6/C7	27°	43°	35°
C7/Th1	15°	64°	14°
Th1/Th2	19°	66°	-
Th2/Th3	15°	44°	15°
Th3/Th4	21°	31°	15°
Th4/Th5	11°	38°	-
Th5/Th6	-	43°	-
Th6/Th7	-	53°	17°
Th7/Th8	17°	57°	3°
Th8/Th9	2°	81°	7°
Th9/Th10	12°	56°	3°
Th10/Th11	-	37°	1°
Th11/Th12	9°	-	30°
Th12/Th13	10°	-	32°
Th13/L1	-	-	32°
L1/L2	36°	16°	33°
L2/L3	24°	24°	26°
L3/L4	22°	19°	28°
L4/L5	20°	50°	18°
L5/L6	-	38°	22°
L6/S1	14°	31°	18°

**Table 2:** Examination of cow 1.

No. of the Appointment	Observation of the Stable Staff	Examination in the Stand	Visual Gait Analysis	Chiropractic Findings
1	slight kicks with right hind limb when standing and walking	slight kyphosis of the dorsal line, kicks out backwards with the right hind limb after getting up, hypermobility in the thoracolumbar transition, restricted lateroflexion of the lumbar spine, reduced weight bearing on the right hind limb	low-grade kyphosis of the back, low-slung head, stiff back and lack of pelvic rotation, LS 3	C5 BR, Th3 PL, Th6 PL, 10th rib P on both sides, L6 PR, PI Ilium L
2 (after 3 weeks)	no kicks when standing, but when walking only on slippery surfaces	slightly raised back line, slight hypermobility in the thoracolumbar transition, limited lateroflexion of the lumbar spine, evenly loaded hind limbs	low-grade kyphosis, severely restricted pelvic rotation and lateroflexion of the lumbar spine, normal head posture, LS 3	C3 BL, Th3 P, Th4 PR, L6 PR, L5 P, L3 PR, AS Ilium R
3 (after 4 weeks)	cows are on pasture, no kicking on the pasture	minor restriction of neck extension, slightly raised dorsal line, no cramping on pasture	slightly raised back line, no cramping on pasture but slight kicks with right hindlimb on a slippery surface in the stable, severely restricted pelvic rotation and lateroflexion of the thoracic spine, slight lameness of the right forelimb, LS 3	C0 SR, Th7 PL, Th5 PL, Th8 P, Th11 P, mobilisation along the lumbar spine
4 (after 9 weeks)	clear further improvement	little kick of the right hind limb after getting up	no kicks, slight kyphosis of the back line, moderately restricted pelvic rotation, slight hypermobility of the thoracolumbar transition, LS 2	C6 BL, Th3 PL, Th4 PR, L1 PR, L5 PR, PI Ilium R, AS Ilium L

**Table 3:** Examination of cow 2.

No. of the Appointment	Observation of the Stable Staff	Examination in the Stand	Visual Gait Analysis	Chiropractic Findings
1	toe tip ulcer of boths hind limbs two months ago; since then, partial tremor of the hind limbs	positioning the hind limbs slightly more under the abdomen, slight kyphosis	moderate kyphosis, slight lameness of both hind limbs during the late stance phase, LS 3.5	Th9 PR, L4 PL, PI Ilium R
2 (after 2 weeks)	improvement one week after treatment	normal distributed load on the hind limbs	restricted rotation in thoracic spine, slight lameness of the right hind limb (during stance phase), slight kyphosis, hypermobility of the sacrolumbar transition, LS 3	Th4 PR, L5 PR
3 (after 3 weeks)	got better within one week after treatment; since then, without symptoms	-	-	-

**Table 4:** Examination of cow 3.

No. of the Appointment	Observation of the Stable Staff	Examination in the Stand	Visual Gait Analysis	Chiropractic Findings
1	sudden lameness in the last 3–4 months despite claw health, kicks out backwards when walking and when standing in the milking parlour, increases with excitement	kicks out backwards when shifting weight from one side to the other and when agitated, dorsoventral flexion of thoracic and lumbar spine moderately restricted, poorly muscled	kicks out backwards when starting to walk and when changing tempo, moderate lordosis of sacrolumbar region, and kyphosis of thoracic spine, short steps, LS 3	C1 PL, C5 BL, Th2 P, Th13 P, 12th rib PL, L4 P, AS Ilium R, 1st–3rd coccygeal vertebrae restricted, very much mobilisation in thoracic and lumbar spine necessary
2 (after 3 weeks)	no kicks of the hind limbs the first three days after treatment, then again in the milking parlour and under stress (herding) as shown before the first treatment; in group (free movement), more “stable”/less kicks	clear improvement in the mobility of the thoracic spine, mild stiffness of the lumbar spine, longissimus dorsi muscle (lumbar); gluteal muscles on the right side moderately warm and increased in size	mild lordosis of sacrolumbar region, backward kicks only in transition from stand to walk, more active hind limb during walk than usually shown in cattle, short steps, LS 2.5	L6 PL, AS Ilium R
3 (after 2 weeks)	no kicks of the hind limbs the first two days after the last treatment, then again with the right hind limb and less frequently with the left, clearly less frequently in the milking parlour	mild nodular tension of the right gluteus region (multiple foci)	right hind limb seldomly kicks backwards during herding, moderately limited pelvic rotation, regular movement in the back line, spacious (normal) steps, LS 2	C7 BR, Th10 PR, Th11 PL, L6 P, S-A L
4 (after 3 weeks)	only right hind limb kicks rarely, further improvement when standing in the milking parlour	moderate oedema in the right gluteal region, mild oedema in the left gluteal region, mild nodular tension of the longissimus dorsi muscle (lumbar, more on the right side than left)	right hind limb seldomly kicks backwards during herding, LS 2	Th2 PR, Th8 P, L3 P, PI Ilium R
5 (after 4 weeks)	cows are on pasture, no kicking on the pasture, only right hind limb kicks rarely (equivalent to about 20% of the initial symptoms)	mild oedema in the right gluteal region, mild nodular tension in the right gluteal region	right hind limb seldom kicks backwards on a slippery surface in the stable, no symptoms on the pasture (lame-free, even and regular gait), LS 1.5	C6 BL, Th6 PL, Th10 PR, Th11 P, L3 PL, S-B P, AS Ilium R
6 (after 9 weeks)	no symptoms	-	-	-

**Table 5:** Examination of cow 4.

No. of the Appointment	Observation of the Stable Staff	Examination in the Stand	Visual Gait Analysis	Chiropractic Findings
1	lameness	less weight bearing of the right hind limb	slight lameness of the right hind limb, no pelvic movement, LS 2.5	C3 BR, C6 BR, Th5 P, Th6 PR, L4 PL, L5 P, S-A L, PI Ilium R
2 (after 3 weeks)	clear improvement, calmer in the milking parlour, turns right hind limb slightly outwards when walking	normal distributed load on the hind limbs	minimal lameness of the right hind limb, regular movement of the back and pelvis, LS 2.0	10th rib RP, L3 P, AS Ilium R
3 (after 2 weeks)	clear improvement, increased toe sensibility after claw trimming	-	-	-
4 (after 3 weeks	clear further improvement	-	-	-
5 (after 4 weeks	normal gait	-	-	-

**Table 6:** Examination of cow 5.

No. of the Appointment	Observation of the Stable Staff	Examination in the Stand	Visual Gait Analysis	Chiropractic Findings
1	lameness and restlessness in the milking parlour	chiropracticly uncorrectable pelvic obliquity	slight lameness of the right hindlimb (shortened protraction phase and abduction), LS 2.5	C5 BR, Th8 PR, L3 PR, PI Ilium R
2 (after 3 weeks)	calm in the milking parlour	same findings	same findings	Th8 PR

## Data Availability

All data generated or analysed during this case are included in this article. Further enquiries can be directed to the corresponding author.
